# Clinical outcomes and quality of life of patients after surgical treatment of a tibia plateau fracture

**DOI:** 10.1007/s00068-025-02963-y

**Published:** 2025-10-28

**Authors:** Lina F. Höller, Sebastian Höller, Elias Klinger, Danny Henkies, Andreas Leha, Wolfgang Lehmann, Corinna C. Dobroniak, Daniel B. Hoffmann

**Affiliations:** 1 Department of Trauma, Orthopedic and Plastic Surgery, University Medical Center Göttingen (UMG), Göttingen, Germany; 2 Scientific Core Facility for Medical Biometry and Statistical Bioinformatics (MBSB), University Medical Center Göttingen (UMG), Göttingen, 37075 Germany

**Keywords:** Tibial plateau fracture, Surgical treatment, Clinical outcomes, Quality of life, Lysholm score, Insurance status

## Abstract

**Purpose:**

Tibial plateau fractures are serious and challenging injuries of the knee joint, leading to severe long-term complications and permanent functional impairment of the knee joint. The aim of this study was to evaluate the outcome and quality of life after tibial plateau fracture and possible influencing factors.

**Methods:**

This retrospective cohort study included patients who underwent surgical treatment for tibial plateau fractures between 2017 and 2020 at the University Medical Center, Göttingen. The endpoints of this study were clinical outcomes, complications, length of hospital stay, sporting ability, and the ability to work. Furthermore, quality of life (SF-36 questionnaire), everyday function (Lysholm Knee Score), subjective self-assessment of satisfaction, and the influence of health insurance status were analyzed.

**Results:**

A total of 117 patients were included, 55 of whom completed the questionnaires. The mean length of hospital stay was 21 (± 17) days. The most common postoperative complications were wound infection and pseudarthrosis (7.7%). Of the 55 patients who completed the questionnaire, 85.7% regained their ability to work after 28 weeks of incapacity for work. Sporting ability was regained in 31% of the 55 patients. Whereas the subjective satisfaction in our patients was good (56.6% satisfied, 20.8% rather satisfied), the result of the Lysholm score was “poor” on average (mean 63 ± 27) and the SF-36 scores were worse on average compared to the normal population. A correlation between lower BMI and a better outcome was shown by better results in both the Lysholm score and SF-36 dimension (physical functioning).

**Conclusion:**

Our study could underline that fractures of the tibial plateau are still an injury that have major impact on the quality of life of those affected. We were able to determine increased body weight as a parameter with a negative influence on physical function scores and psychological and emotional well-being. The clinical results were independent of health insurance status in Germany.

## Introduction

Tibial plateau fractures are serious and challenging injuries of the knee joint, predominantly affecting younger people during their most active years, frequently leading to severe and long-term complications and permanent functional impairment of the knee joint [1–4].

Road traffic accidents and other high-energy trauma are among the most common causes of tibial plateau fractures, accounting for more than 50% of the cases [5, 6]. Due to the accident mechanisms and the anatomical conditions of the knee joint, tibial plateau fractures are often associated with concomitant injuries [4]. The tibial plateau bears a significant portion of the physical load and is exposed to more constant and regular static and dynamic stresses than other joints [7–9]. The timing and type of treatment are particularly relevant to the outcomes [10]. Depending on the extent of the fracture, the surgical treatment of tibial plateau fractures can be very complex and continues to pose a challenge for medical staff [11, 12]. The number of intraoperative and postoperative complications is correspondingly high, both in the past and today, up to 20% [6, 11, 13, 14].

Tibial plateau fractures remain difficult to treat, with suboptimal and unsatisfactory treatment outcomes of up to 50% [8, 15, 16]. Many patients still experience moderate outcomes owing to persistent complaints and functional impairments. Many patients experience reduced quality of life and impaired ability to work [3, 6]. The main causes are complications of wound healing, infections, and development of secondary knee joint arthrosis, resulting in loss of mobility [4, 17].

Optimal care with as few complications as possible would be extremely important for the outcome of each individual, but also for our society, because a lot of the workforce is lost due to poor outcomes. However, there are inconsistent treatment recommendations and few studies on this topic [7, 18]. Data on the quality of life experienced by patients and the factors influencing quality of life are missing.

The aim of this study was to analyze the clinical outcomes and quality of life of patients after tibial plateau fractures and determine the factors influencing these outcomes.

## Methods

This retrospective cohort study included patients who underwent surgical treatment for tibial plateau fractures between 2017 and 2020 at the University Medical Center Göttingen. Additionally, the patients were followed up and interviewed using questionnaires. This study was approved by the local ethics committee (study number 4/11/22). All experiments were performed in accordance with the principles of the Declaration of Helsinki.

Patients were eligible for inclusion if they were older than 18 years, were primarily treated at the University Medical Center Göttingen, and voluntarily decided to participate in the study on informed consent after receiving the questionnaires. In addition to patients who had not reached the age of 18 years at the time of primary treatment, patients who had undergone initial surgical treatment in another hospital and were only at the University Medical Center Göttingen for follow-up examinations or follow-up treatments (such as metal removal) were not included.

Electronic medical records were used to identify patient demographics and comorbidities. Baseline characteristics (age and sex), risk factors, and comorbidities such as diabetes mellitus, smoking, and body mass index (kg/m^2^) were recorded. In addition, the insurance status (statutory health insurance, private health insurance, employers’ liability insurance associations (German: “Berufsgenossenschaft/BG”). Second, preoperative and operative characteristics (such as preoperative imaging, classification of the fracture, and type of operation) and clinical outcomes (such as complications, reinterventions, and length of hospital stay) were recorded. Finally, the patients were followed up, and data on functional impairment of the knee joint, pain, quality of life, everyday function, ability to work, and development of post-therapeutic sports abilities were collected. Therefore, the patients were interviewed using questionnaires that included the Lysholm and SF-36 scores.

The endpoints of this study were clinical outcomes, complications, length of hospital stay, sporting ability, and the ability to work. Furthermore, quality of life (SF-36 questionnaire), everyday function (Lysholm Knee Score), and subjective self-assessment of satisfaction were analyzed. The Lysholm Knee Score is a questionnaire comprising eight items, designed to assess the degree of knee instability at both the levels of impairment and limitation. A high Lysholm score corresponds to a low degree of knee instability.

Standard methods were used for descriptive statistics in this study. Categorical data were presented as percentages, and continuous data as means combined with standard deviation (SD). Statistical significance was set at *P* < 0.05. significant. Differences between independent groups were assessed using the Brunner–Munzel, Jonckheere-Terpstra, and rank-based tests. Additonally, Fisher’s Exact Test for count data, Cochran-Armitage test for trend, Pearson’s product-moment correlation, Kendall’s rank correlation tau, analysis of variance, Welch’s two-sample t-test, and studentized permutation test were applied. All analyses were performed using the R statistical software (version 4.1.1; R Core Team 2021). The R package lme4 (version 1.1.33; Bates et al. 2015) was used for logistic regressions with mixed effects. All statistical analyses were performed in cooperation with the Scientific Core Facility for Medical Biometry and Statistical Bioinformatics at the University Medical Center, Göttingen.

## Results

A total of 117 patients aged 19–90 years were included in this study. The mean age of all patients was 49 years. Within the patient group, 60.7% of all patients were male and 39.3% were female. The average body mass index of the patients was 28 kg/m² (± 6 kg/m²). Most patients (55.4%) had statutory health insurance; 10.7% of the patients were members of a private health insurance system. Of the accidents, 33.9% occurred during work and were therefore insured by employers’ liability insurance associations. The results of the analyses of other comorbidities and risk factors are presented in Table 1.

In our study, 62.1% of patients had a tibial plateau fracture due to high-energy trauma, mostly in the form of road traffic accidents (34.8%), followed by sports accidents, falls, and accidents at work. The most common fractures were AO type C (48.6%) and B (45%) fractures. In terms of trauma-related concomitant injuries, soft tissue damage was the most common in 63 patients (53.8%). Not all patients routinely undergo preoperative magnetic resonance imaging (MRI). In patients who underwent magnetic resonance imaging, the most commonly detected concomitant Ligament damage was rupture of the anterior cruciate Ligament in 8.5% of all cases, followed by confirmed rupture of the lateral collateral Ligament in 5.1%, rupture of the medial collateral Ligament in 3.4%, and rupture of the posterior cruciate Ligament in 2.6%. Meniscal damage was confirmed in 16.2% of patients.

**Table 1:** Patient demographics, comorbidities and fracture classification

	Patients (*n* = 117)
**Mean age (years)**	49 (± 16)
**Gender**	
Male	71 (60.7%)
Female	46 (39.3%)
**Insurance status**	
Statutory health insurance	65 (55.4%)
Private health insurance	13 (10.7%)
Employers’ liability insurance associations	39 (33.9%)
**Comorbidities**	
BMI (kg/m²)	28 (± 6)
Smoking	24 (20.5%)
Cardiovascular diseases	44 (37.6%)
Hypo- or hyperthyroidism	15 (12.8%)
Osteoporosis	10 (8.5%)
Diabetes mellitus	6 (5.1%)
**AO classification**	
Type A	7 (6.3%)
Type B	53 (45.3%)
Type C	57 (48.6%)

Data are presented as mean + standard deviation (for continuous data) or percentage (for categorical data)

BMI = body mass index

Regarding surgical care, 39 patients (33.9%) were fitted with an external fixator for primary care. Of these, 87.2% received an external fixator on the day of the accident, 10.3% on the following day, and one patient on the 7th day after the trauma. The average time interval until the final surgery was 12 (± 8 days). In the final surgical care, 55.8% of tibial plateau fractures were treated with one osteosynthesis plate, 27.4% with two plates, and 4.4% with three osteosynthesis plates. Screw osteosynthesis alone was used in 8.5% of patients. One patient underwent primary knee joint arthroplasty and another patient received an arthrodesis nail. Single surgical access was used in 69.3% of the operative treatments. A total of 29.7% of the operations were performed with two surgical approaches and 1% with three surgical approaches. Most patients underwent surgery using only the lateral approach (57.4%), followed by a combination of lateral and medial approaches (20.8%). Details of the operative characteristics are presented in Table 2.

**Table 2:** Operative characteristics

	Patients (*n* = 117)
External fixator	39 (33.9%)
Time interval until final surgery (days)	12 (± 8)
**Type of operation**	
One osteosynthesis plate	65 (55.8%)
Two osteosynthesis plates	32 (27.4%)
Three osteosynthesis plates	5 (4.4%)
Screw osteosynthesis	10 (8.5%)
Athrodesis nail	1 (0.9%)
Primary knee joint arthroplasty	1 (0.9%)
Intramedullary nail	1 (0.9%)
Kirschner-wire osteosynthesis	1 (0.9%)
Screw + Kirschner-wire osteosynthesis	1 (0.9%)
**Surgical approach**	
Lateral approach	67 (57.4%)
Medial approach	13 (10.9%)
Lateral + medial approach	24 (20.8%)
Lateral + dorsal approach	8 (6.9%)
Medial + dorsal approach	2 (2%)
Lateral + medial + dorsal approach	1 (1%)
Dorsal approach	1 (1%)

Data are presented as mean + standard deviation (for continuous data) or percentage (for categorical data)

### Clinical endpoints

The average length of hospital stay was 21 days (± 17 days); the average time interval until final surgical care was 12 days (± 8 days). The most common postoperative complication was a wound infection (7.7%). Of the 117 patients, 55 completed and returned questionnaires. This corresponded to a response rate of 47%. The questionnaires revealed that, on average, after 28 weeks of incapacity for work, 85.7% of the patients regained their ability to work. There was no significant difference in the duration of work incapacity between accidents during work (insurance status: employers’ liability insurance associations) and accidents insured differently (insurance status: statutory or private health insurance) at 31 and 25 weeks. Sporting ability was regained by 31% (Table 3). During follow-up, 18.8% of patients developed signs and symptoms of secondary gonarthrosis. The mean follow-up duration was 31 months.

**Table 3:** Clinical endpoints

	Patients (*n* = 117)
Length of hospital stay (days)	21 (± 17) (range 4–119)
Complications	
Wound infection	9 (7.7%)
Dislocation of the osteosynthesis material	3 (2.6%)
Thrombosis	4 (3.4%)
Arthrofibrosis	4 (3.4%)
Peroneal lesion	2 (1.7%)
Secondary gonarthrosis	22 (18.8%)
Postoperative rehabilitation	87 (74.4%)
	Patients (*n* = 55)
Sporting ability	17 (31%)
Ability to work	47 (85.7%)
Duration of incapacity for work (weeks)	28 (± 20)
Permanent reduction in gainful employment	20 (36.6%)

Data are presented as mean + standard deviation (for continuous data) or percentage (for categorical data)

**Fig. 1:**
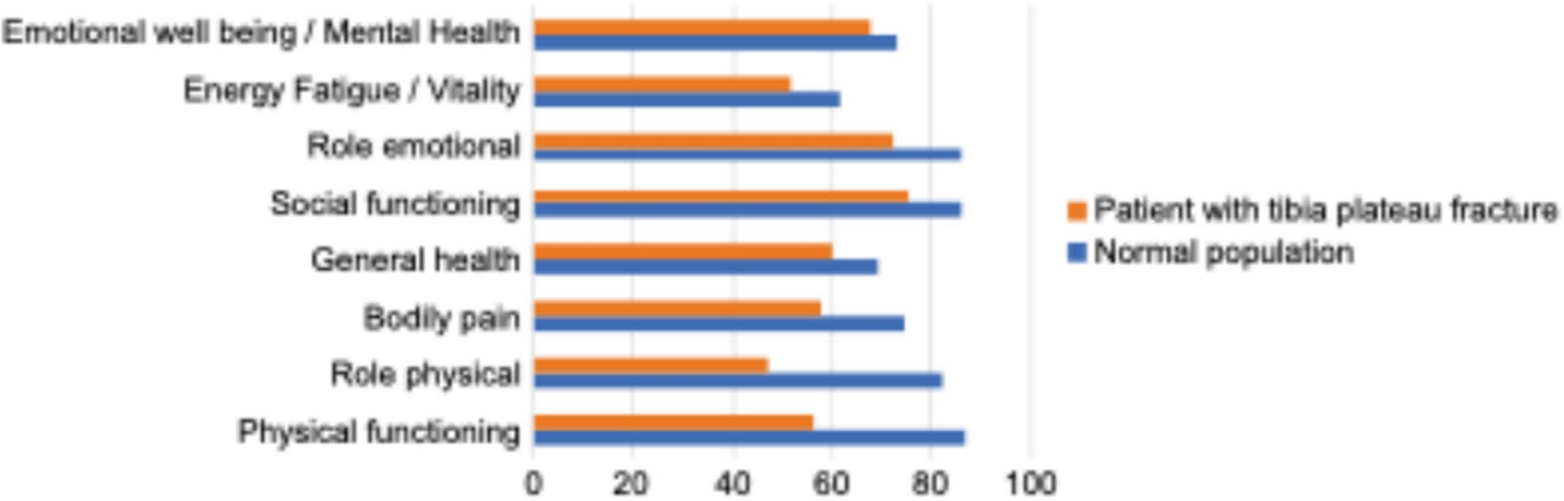
SF-36: Comparison with the normal population

The results regarding the endpoints of quality of life (SF-36 questionnaire), everyday function (Lysholm and Gillquist questionnaire), and subjective self-assessment of satisfaction are shown in Table 4. Whereas the subjective satisfaction in our patients was good (56.6% satisfied, 20.8% rather satisfied), the result of the Lysholm score was “poor” in most of the cases (52.7%). More than 60% with a “poor” Lysholm score had an AO type C fracture. Patients with Type C fracture showed in 56.7% a “poor”, in 20% a “fair”, in 10% a “good” and in 13.3% an “excellent” score (Table 5). Whereas patients with Type B fracture showed in 45.5% a “poor”, in 13.6% a “fair”, in 18.2% a “good” and in 22% an “excellent” score (Table 5). Only 2 patients with Type A fracture responded the Lysholm questionnaire. Interestingly both showed only a “poor” or “fair” score. However, in statistical analysis the influence of fracture classification on the Lysholm was not significant. The results for the eight dimensions of SF-36 are presented in Table 4. We could see that the patients’ score was worse on average in comparison to the normal population, particularly in the dimensions “Role limitations due to physical health” and “Physical functioning” (Fig. 1).

**Table 4:** Quality of life, everyday function and subjective self-assessment of own satisfaction data are presented as mean + standard deviation (for continuous data) or percentage (for categorical data)

	Patients (n = 55)
**Subjective self-assessment of own satisfaction**	
Satisfied	31 (56.6%)
Rather satisfied	11 (20.8%)
Less satisfied	9 (17%)
Unsatisfied	3 (5.7%)
**Lysholm score**	
Mean score (range 0-100 points)	63 (± 27)
Poor (< 64 points)	29 (52.7%)
Fair (65–83 points)	10 (18.2%)
Good (84–94 points)	7 (12.7%)
Excellent (95–100 points)	9 (16.4%)
**SF-36**	
Emotional well being/mental health	68 (± 21)
Energy fatigue/vitality	52 (± 22)
Role limitations due to emotional problems	72 (± 42)
Social functioning	75 (± 28)
General health	60 (± 22)
Bodily pain	58 (± 29)
Role limitations due to physical health	47 (± 45)
Physical functioning	56 (± 33)
Health change	51 (± 25)

Data are presented as mean + standard deviation (for continuous data) or percentage (for categorical data)

**Table 5:** Lysholm score related to AO classification

Lysholm score	Poor	Fair	Good	Excellent
Type A	1 (50%)	1 (50%)		
Type B	10 (45,5%)	3 (13.6%)	4 (18.2%)	5 (22%)
Type C	17 (56,7%)	6 (20%)	3 (10%)	4 (13.3%)

### Outcome-influencing factors

No correlation was found between patient body mass index (BMI) and fracture complexity. Primary treatment with an external fixator was performed in 33.9% of cases; the application of an external fixator had no significant influence on perioperative infections. Continuous observation of the dependence of the Lysholm score on the patient’s BMI using Pearson’s product-moment correlation showed that the Lysholm score decreased with increasing BMI. This resulted in a statistically significant correlation coefficient of −0.341 (*p* = 0.012). In contrast, the fracture classification and performance of postoperative rehabilitation on the other hand had no statistically significant influence on the Lysholm score in our study. In addition, insurance status had no significant influence on Lysholm scores.

Regarding the SF-36 questionnaire, the two dimensions were also significantly influenced by patients’ BMI. The SF-36 scores (physical functioning) decreased significantly with increasing BMI (*p* = 0.032). Pearson’s productmoment correlation showed a correlation coefficient of −0.29. Furthermore, the SF-36 score (role limitations due to emotional problems) showed a statistically significant correlation with the existing BMI at the time of injury (*p* = 0.009). The score values showed significantly worse results, that is, a higher degree of limitations due to emotional problems with increasing body mass index. The values show a clear negative correlation coefficient of −0.2858 for Kendall’s rank correlation, tau. Fracture classification and performance of postoperative rehabilitation, on the other hand, had no statistically significant influence on the SF-36 dimensions, physical functioning, or “role limitations due to emotional problems”. The SF-36 dimensions “Role limitations due to physical health,” “Energy fatigue,” “Emotional well-being,” “Social functioning,” “Pain,” “General health” and “Health change” were not significantly influenced by any of the three factors BMI, fracture classification and postoperative rehabilitation.

## Discussion

Tibial plateau fractures are serious and difficult to manage, frequently leading to severe long-term complications and permanent functional impairment of the knee joint [1–4]. This study aimed to evaluate the treatment of patients with a tibial plateau fracture by analyzing the clinical outcomes and rare data on the quality of life of patients.

Of the 117 patients retrospectively reviewed, 55 completed and returned the questionnaires. This corresponded to a response rate of 47%. This value is in the upper range of the average post-survey response rate of 45% [19]. We assessed physical and subjective outcomes using the Lysholm and SF-36 scores, respectively. These are the two established scores recommended by the guidelines for the follow-up assessment of tibial plateau fractures.

We showed that many highly complex tibial plateau fractures were treated at our institution because most of the fractures were type C (48.6%), followed by type B (45%). Type A fractures were a minority (6.3%) because we only included surgically treated fractures and type A fractures were more likely to be suitable for conservative treatment. In comparison to the literature, our proportion of type C fractures is much higher, most likely because our institution is a supra-regional maximum care provider to which patients are primarily referred for more complex injuries [8, 20].

The results underline that the treatment of these complex patients was quite successful in most patients: 85.7% regained their ability to work, 31% regained their ability to participate in sports, and 77.4% of the patients provided a positive statement regarding their personal assessment of their satisfaction. A negative aspect of our results was the overall complication rate of 18.8% (22/117), with most complications being wound infections. This complication rate was slightly higher than those reported in the literature. Le Baron et al. (2019) reported complication rates of 8–9% and Kugelman et al. (2017) reported an overall complication rate of 16% [21, 22]. The high number of wound healing problems and infections in our group of patients was most likely due to complex injuries, with a high rate of initial soft tissue damage due to the accident. In particular, more complex fractures are more frequently associated with severe soft tissue damage and are therefore a favorable basis for wound infections [23]. In our study, 63 patients (53.8%) initially reported soft-tissue damage, which is a risk factor for infection, delayed wound healing, and pseudarthrosis. Furthermore, undetected low-grade infections according to prolonged wound healing and wound infections can be a problem in back-up strategies such as arthroplasty or proximal tibial replacement. Further studies are needed to clarify when a reconstruction of tibial plateau fractures with significant soft-tissue damages is recommended and when its recommended to wait for a proper arthroplasty.

Additionally, 33.9% of our patients received an external fixator as their first surgical care. This also poses the risk of introducing an infection with the subsequent risk of osteomyelitis, material loosening, and/or the need to change the material [24]. However, no direct correlation was observed in the present study.

Our results showed that the patients’ personal assessments of their satisfaction with the therapy were generally good. Most patients (56.6%) were “satisfied” and 20.8% were “rather satisfied.” Only 17% were “less satisfied” and 5.7% state that they were “dissatisfied.” Surprising is the simultaneous result of the Lysholm score, which was with a mean of 63 points out of possible 100 points in the category “poor” outcome. This score is comparable with the results of Roßbach et al. (2016) and Jansen et al. (2013) [3, 6]. Better scores were found in Wilde (2010) and Attmanspacher et al. (1998), with average scores of 81 and 84 points [25, 26]. Kraus et al. (2012) reported a significantly higher score of 76.6 points [20]. Even if we could show, that more than 60% of patients with “poor” outcome had an AO type C fracture, it is a Limitation in the present study is, that we cannot respond to the question, if a worse outcome and quality of Life is based on an incomplete reduction or persisted articular step of more than 2 mm after surgery. In general, control of reduction after surgery was done in this study by x-ray. A CT scan after surgery was done only in some cases. From the authors point of view a CT scan for control after surgery is recommended in future in general for tibial plateau fractures.

Based on our Lysholm scores, one would intuitively expect a significantly worse result for subjective satisfaction with the final outcome. However, the distribution of the Lysholm score in “Poor” to “Excellent” is inversely proportional to the subjective results of treatment satisfaction. This raises the question of the influence of post-therapeutic activity and functional ability on patient well-being. Other factors, such as psychological well-being, have a greater influence on the quality of life than previously assumed. This should be investigated in detail in future studies. In our study in Germany, the clinical results were independent of health insurance status. There was also no significant difference in the duration of work incapacity. The existing literature on the influence of health insurance status on the outcomes of surgical treatment in Germany is generally very poor. To the best of our knowledge, no previous study has included patients with tibial plateau fractures. In the United States, few studies have analyzed the impact of health insurance status on the outcome of orthopaedic treatments, including shoulder arthroplasty and total knee arthroplasty, describing a lower risk of perioperative medical and surgical complications for patients with a private insurance payment status compared to age- and sex-matched patients with Medicare and Medicaid/uninsured payer status [27–29]. In Germany, more attention should be paid to the healthcare status of patients included in future studies.

In addition, our results showed that a normal body mass index correlated with better physical outcomes. Significance between lower BMI and the outcome was shown in a better result in the Lysholm score, the activity level of those affected, and in the SF-36 dimension “Physical functioning.” The correlation of BMI with the “Role limitations due to physical health” and “Pain” dimensions of the SF-36 were not statistically significant but show a similar trend. Similar results were reported by Dinçel et al. [18]. They also show a negative effect of increased BMI and a positive effect of decreased BMI on functional outcomes [18]. Furthermore, a BMI within the normal weight range had a significantly positive influence on psychological aspects. Those affected stated that they had significantly fewer emotional problems, as measured by the SF-36 dimension ‘Role limitations due to emotional health’. Therefore, there seems to be a correlation between increased BMI and increased levels of emotional problems in terms of the quality of life. In general, several studies have shown that the quality of life in both physical and psychological contexts decreases with increasing body mass index [30–32]. Consequently, the body mass index is definitely an aspect that seem to influence the outcome of surgical care of tibia plateau fractures. However, a study conducted by Alazzawi et al. (2011) investigated the correlation between body weight and the long-term outcome of fractures of the lower extremities and found no correlation between body weight and fracture complications or fracture healing [33]. Therefore, it is possible that the negative effects on physical functioning measured in our study were due to obesity itself, and not due to the negative effects of obesity on fracture outcomes. In contrast, McGurk et al. (2023) reported that increased BMI is associated with increased fracture complexity and an increased rate of post-operative complications, including wound infections, venous thromboembolism, and wound dehiscence [34]. However, our results suggest that mental well-being should be monitored throughout treatment and addressed in appropriate situations so that existing offers can be made if necessary.

In our study, no correlation was found between the fracture classification and outcome, which is consistent with previous studies. The results in the literature are mixed als well. Some studies have shown that patients with type C fractures or higher-grade fractures show worse results than those with type A, type B, and generally lower-grade fractures [35–37]. Similar results were observed for the Lysholm score. Kraus et al. (2012) showed that bicondylar type C fractures achieved significantly fewer points on the Lysholm score than type A and B fractures [20]. However, some studies have not found a significant influence of fracture complexity on functional outcomes [1, 18, 38]. Therefore, it is not possible to base a forecast on the final outcome of the fracture classification according to the AO alone.

We could see that the patients’ score within all eight SF-36 dimensions was on average worse in comparison to the normal population, particularly in the dimensions “Role limitations due to physical health” and “Physical functioning.” The comparative data comes from the “Study on the Health of Adults in Germany” (DEGS), which was published by the Robert Koch Institute and provides health-related data from 2008 to 2011 [39]. This underlines the impact of the surgical treatment of tibial plateau fractures on patient functioning and well-being.

## Limitations

The following Limitations of this study should be taken into account when interpreting and discussing the results. Due to the retrospective design, recall bias and missing data may have affected the outcomes. Furthermore, there may have been bias in the responses to the questionnaires in this prospective study. Patients with better outcomes after an injury are often more satisfied and, more willing to participate in post-therapeutic surveys. In this respect, it is conceivable that patients with better outcomes are more Likely to respond to questionnaires. The case numbers of 117 and 55 patients, respectively, made it difficult to obtain statistically significant statements. Furthermore, the aspect of chance must be considered and assessed to obtain significant results with a small number of cases. Another limitation in the present study is, that we cannot respond to the question, if a “poor” outcome and quality of Life is based on an incomplete reduction or persisted articular step of more than 2 mm after surgery. The analyses of potential risk factors were conducted with a screening character and were intended as hypothesis generating. Therefore, the presented P-values have not been adjusted for multiple testing and should be interpreted as descriptive only. Not all concomitant injuries were always recognized with certainty because a magnetic resonance imaging was not routinely performed in all cases.

## Conclusion

In conclusion, our study could underline that fractures of the tibial plateau still have major impact on the quality of life of those affected. We found increased body weight as a parameter that negatively affects physical function scores and psychological and emotional well-being. The clinical results of our study were independent of insurance status in Germany.

## Data Availability

The data that support the findings of this study are not openly available due to reasons of sensitivity and are available from the corresponding author upon reasonable request.
