# Publisher Correction: Clinical outcomes and quality of life of patients after surgical treatment of a tibia plateau fracture

**DOI:** 10.1007/s00068-026-03154-z

**Published:** 2026-03-17

**Authors:** Lina F. Höller, Sebastian Höller, Elias Klinger, Danny Henkies, Andreas Leha, Wolfgang Lehmann, Corinna C. Dobroniak, Daniel B. Hoffmann

**Affiliations:** 1 Department of Trauma, Orthopedic and Plastic Surgery, University Medical Center Göttingen (UMG), Göttingen, Germany; 2 Scientific Core Facility for Medical Biometry and Statistical Bioinformatics (MBSB), University Medical Center Göttingen (UMG), 37075 Göttingen, Germany

**Publisher Correction: European Journal of Trauma and Emergency Surgery (2025) 51:319**

10.1007/s00068-025-02963-y

The original version of this article, published on 28 October 2025, unfortunately contained a mistake.

During the typesetting process, an error occurred in the display of Figure 1. This error was only visible in the PDF; the online version of the article was correct.

The original article has been corrected.

The publisher apologizes for the error.

